# Supplementary material for: An in silico framework for the rational design of vaginal probiotic therapy
Source: PLoS Comput Biol. 2025 Feb 14;21(2):e1012064. doi: 10.1371/journal.pcbi.1012064 (PMC11867318; doi:10.1371/journal.pcbi.1012064)
Supplement: S1 Table — Note the determination of inter-species interaction terms was based on empirical observation and hypothesis on interaction term strength and directionality. More information can be found in a previous publication in Supplementary Note 1 [42]. Values were scaled to in vivo rates based on Stein et al. [37]. This table also includes references [71–75]. (DOCX) [file pcbi.1012064.s001.docx]

**S1 Table.**

| **Parameter**  (*in vitro/in vivo*) | **Value** | **Explanation** | **References** |
| --- | --- | --- | --- |
| k_grow-nAB_ (hr^-1^)/(d^-1^) | 0.1 to 1.00 | Growth rate calculated in previous publication from growth curves (Lee et al., 2020)[71] and assessed from digitized growth curves from literature such as Atassi et al., 2019[15] and Anukam and Reid (2008)[72]. | [19,71,72] |
| k_grow-Li_ (hr^-1^)/(d^-1^) | 0.1 to 1.00 | Growth rate calculated in previous publication from growth curves (Lee et al., 2020)[71] and from doubling times (Borgogna et al., 2021)[41]. | [41,71] |
| k_grow-oLB_ (hr^-1^)/(d^-1^) | 0.1 to 1.00 | Growth rate calculated in previous publication from growth curves (Lee et al., 2020) and from digitized data in Chetwin et al., 2019[73] and Juarez-Tomas (2003)[74] as well as Borgogna et al (2021)[41]. | [41,71,73,75] |
| α_nAB→ nAB_, α_Li → Li_, α_oLB → oLB_  (hr^-1^cell density^-1^)/ (d^1^cell density^-1^) | -0.004 to  -0.04 | Assumed similar carrying capacities are possible across species and a 10-fold variability. This value and the growth rate value facilitate up to 100-fold variation in carry capacity and clinically relative abundance can upwards of range 1000-fold. | [12,13,43] |
| α_nAB → Li,_ α_nAB → oLB,_ α_Li → nAB,_ α_Li → oLB,_ α_oLB → Li_  (hr^-1^cell density^-1^)/ (d^1^cell density^-1^) | -0.12 to  0.12 | Assumed directionally of these parameters to be positive or negative dependent on the literature. Magnitude of the values was determined from the largest ratio of interaction term to self-interact term observed experimentally (S1 Fig). The fold-ratio is on the same order of magnitude as clinically estimated gLV terms (Stein et al. 2013). | [37,53,54] |
| α_oLB → nAB_  (hr^-1^cell density^-1^)/ (d^1^cell density^-1^) | -0.12 to  0.00 | Assume directionality based on experimental observations that oLB spp. commonly inhibit non-optimal spp. (nAB), such as in Atassi et al. (2006). | [15,53] |
