# Supplementary material for: An in silico framework for the rational design of vaginal probiotic therapy
Source: PLoS Comput Biol. 2025 Feb 14;21(2):e1012064. doi: 10.1371/journal.pcbi.1012064 (PMC11867318; doi:10.1371/journal.pcbi.1012064)
Supplement: S2 Table — This table details various probiotic dosing regimens, including whether pretreatment with antibiotics was administered, the routes of administration (vaginal, oral, or both), the length of each regimen, the specific probiotic strains used, the final results, the bacterial vaginosis (BV) evaluation metrics, and the final end points. The diversity in these probiotic regimens demonstrates the variability in outcomes observed across different clinical trials. This table also includes references [76–94]. (DOCX) [file pcbi.1012064.s002.docx]

**S2 Table.**

| **Probiotic Duration** | **Reference** | **Antibiotic** | **Route** | **Probiotic Regimen** | **Strain** | **Results** | **Metric** | **End Point** |
| --- | --- | --- | --- | --- | --- | --- | --- | --- |
| Acute | Petricevic et al. 2008 [76] | Yes | Vaginal | 7 days | L. casei var. rhamnosus (Lcr35) | Significant | Nugent | 5 weeks |
| Acute | Hemmerling 2010 [77] | Yes | Vaginal | 5 days, 1/wk for 2 weeks | L. crispatus CTV-05 | Significant | Recurrence rate | 28 days |
| Acute | Happel 2020 [31] | Yes | Both | oral or vaginal spray (15 days, 5 days oral followed by 10 days oral + vaginal spray) | L. acidophilus, L. rhamnosus GG, B. bifidum and B. longum | Mixed results |  |  |
| Acute | Ehrstrom 2010 [78] | Yes | Vaginal | 5 days | L. gasseri LN40, L. fermentum LN99, L. casei subsp. rhamnosus LN113 and P. acidilactici LN23 | Not significant | Cure rate |  |
| Acute | Mastro-marino 2009 [32] | No | Vaginal | 7 days | Florisia  (L. brevis CD2 + L. salivarus subsp. Salicinius FV2 + L. plantarum FV9) | Significant | Cure rate, Recurrence rate |  |
| Acute | Bradshaw 2012 [79] | Yes | Vaginal | 12 days | Gynoflor (L. acidophilus KS400 + 0.03 MG ESTRIOL) | Mixed results | Cure rate | 30 days |
| Acute | Hemalatha 2012 [80] | No | Vaginal | 8 days | Florisia  (L. brevis CD2 + L. salivarus subsp. Salicinius FV2 + L. plantarum FV9) | Not significant |  |  |
| Acute | Ling 2013 [81] | Yes | Vaginal | 7 days | *L. delbrueckii* subsp. *Lactis DM8909* | Significant | Reccurence rate | 5 days |
| Acute | Bisanz 2014 [82] | Yes | Vaginal | 3 days | L. rhamnosus GR-1, L. reuteri RC-14 | Not significant |  |  |
| Acute | Verdenelli 2016 [83] | Yes | Vaginal | 7 days | SYNBIO gin (*L. rhamnosus* IMC 501 + *L. paracasei* IMC 502) | Significant | Nugent | 21 days |
| Acute | Rapisarda 2018 | No | Vaginal | 14 days | *L. acidophilus* LA 14 | Significant | Cure rate | 28 days |
| Intermittent | Marcone et al. 2008 [84] | Yes | Vaginal | 2 months (1/wk) | *L. rhamnosus* | Significant |  |  |
| Intermittent | Marcone et al. 2010 [85] | Yes | Vaginal | 6 months (1/wk) | *L. rhamnosus* | Significant |  | 12 mo |
| Intermittent | Heczko 2015 [86] | Yes | Oral | 10 days/month | L. fermentum 57A, L. plantarum 57B, L. gasseri 57C | Significant | Time to recurrence |  |
| Intermittent | Hummelen 2010 [87] | Yes | Oral | 2x/wk, ~6 mnths | L. rhamnosus GR-1, L. reuteri RC-14 | Not significant | Nugent |  |
| Intermittent | Larsson 2008 [88] | Yes | Vaginal | 10days/cycle, 3 cycles | EcoVag (L. gasseri, L. rhamnosus) | Significant |  |  |
| Intermittent | Eriksson 2005 [89] | Yes | Vaginal | 2 cycles | L. fermentum, L. caseivar, L. rhamnosus, and L. gasseri | Not significant | Nugent |  |
| Intermittent | van de Wijgert 2020 [33] | Yes | Vaginal | Intermittent | EF+ (Bifidobacterium bifidum W28, Lactobacillus acidophilus W70, L. helveticus W74, L. brevis W63, L. plantarum W21, L. | Significant | Reduce BV bacteria |  |
| Intermittent | van de Wijgert 2020 [33] | Yes | Vaginal | Intermittent | Gynophilus LP (Lcr regenerans, L. rhamnosus 35) | Significant | Reduce BV bacteria |  |
| Long-term | Bohbot et al. 2018 [90] | Yes | Vaginal | 56 days | *Physioflor (*L. crispatus IP 174178) | Significant | Time to recurrence |  |
| long-term | Martinez 2009 [91] | Yes | Oral | 28 days | L. rhamnosus GR-1, L. reuteri RC-14 | Significant | Cure rate |  |
| long-term | Sudha 2012 [92] | Yes, non-traditional | Oral | 90 days | B. coagulans Unique IS-2 | Significant | Symptoms |  |
| long-term | Anukam 2006 [93] | Yes | Oral | 30 days | L. rhamnosus GR-1, L. reuteri RC-14 | Significant | Cure rate, Recurrence rate | 30 days |
| long-term, intermittent | Cohen 2020 [29] | Yes | Vaginal | 4 days, 2/wk for 10 weeks | L. crispatus CTV-05 | Significant | Recurrence rate | 12 wk, 24 wk |
| long-term, intermittent | Marcotte 2019 [94] | Yes, non-traditional | Vaginal | 30days, 1x/wk 190days | L. rhamnosus DSM 14870, L. gasseri DSM 14869 | Not significant | Cure rate, Recurrence rate |  |
