# Supplementary material for: An in silico framework for the rational design of vaginal probiotic therapy
Source: PLoS Comput Biol. 2025 Feb 14;21(2):e1012064. doi: 10.1371/journal.pcbi.1012064 (PMC11867318; doi:10.1371/journal.pcbi.1012064)
Supplement: S3 Table — The sensitivity of each parameter and response type (nAB, Li, oLB, P-dominant) was measured using a method from previous research, which standardizes the changes in outcomes relative to the variations in parameter values [44]. (DOCX) [file pcbi.1012064.s003.docx]

**S3 Table.**

|  | nAB-dominant |  | Li-dominant |  | oLB-dominant |  | P-dominant |
| --- | --- | --- | --- | --- | --- | --- | --- |
| Parameter | Metric Value |  | Metric Value |  | Metric Value |  | Metric Value |
| α _nAB->P_ | -0.326 |  | -0.13 |  | -0.35 |  | 0.059 |
| α_P->nAB_ | 0.072 |  | -1.028 |  | -0.181 |  | -0.066 |
| α_P->Li_ | 0.021 |  | 0.248 |  | -0.04 |  | -0.059 |
| α_Li->P_ | -0.038 |  | -0.089 |  | -0.031 |  | 0.035 |
| α_P->oLB_ | -0.017 |  | 0.158 |  | 0.302 |  | -0.036 |
| α_oLB->P_ | -0.014 |  | -0.009 |  | -0.158 |  | 0.016 |
| k_grow-P_ | -0.781 |  | -0.018 |  | -0.025 |  | 0.824 |
| α_P->P_ | 0.854 |  | 0.042 |  | 0.049 |  | -0.945 |
