# Supplementary material for: An in silico framework for the rational design of vaginal probiotic therapy
Source: PLoS Comput Biol. 2025 Feb 14;21(2):e1012064. doi: 10.1371/journal.pcbi.1012064 (PMC11867318; doi:10.1371/journal.pcbi.1012064)
Supplement: S4 Table — The Latin Hypercube Sampling (LHS) method was used to generate 500 in silico strains. Among these, the top strains (nAB, Li, oLB, P) were identified as those that elicited one of the four response types in the highest percentage of the virtual population. The Best Fit strains, designated as Latin-V, V2, V3, V4, and V5, were the top 5 strains that most closely matched the performance of L. crispatus CTV-05. This comparison was based on the sum of squares error and was carried out using the same phase 2b Lactin-V regimen, with evaluations conducted at 12 and 24 weeks. (DOCX) [file pcbi.1012064.s004.docx]

**S4 Table.**

|  | k_grow-P_ | a_nAB->P_ | a_Li->P_ | a_oLB->P_ | a_P->nAB_ | a_P->Li_ | a_P->oLB_ | a_P->P_ |
| --- | --- | --- | --- | --- | --- | --- | --- | --- |
| Top nAB | 0.2049 | -0.0465 | 0.11 | -0.0001 | -0.0083 | -0.0963 | -0.1047 | -0.022 |
| Top Li | 0.9489 | 0.1018 | -0.1176 | 0.0994 | -0.0986 | 0.0752 | -0.1042 | -0.022 |
| Top oLB | 0.7106 | 0.1009 | 0.0747 | -0.1124 | -0.0583 | -0.0827 | 0.0937 | -0.022 |
| Top P | 0.5305 | 0.0321 | 0.0994 | 0.0168 | -0.0523 | -0.0068 | -0.0381 | -0.022 |
| Best Fit Lactin-V | 0.253 | -0.101 | -0.0541 | -0.0509 | -0.0617 | -0.0601 | -0.0087 | -0.022 |
| Lactin-V2 | 0.8241 | -0.0174 | -0.1092 | -0.0573 | -0.0072 | -0.0613 | 0.0402 | -0.022 |
| Lactin-V3 | 0.3633 | -0.0148 | -0.0667 | -0.0902 | -0.1043 | -0.0135 | 0.0457 | -0.022 |
| Lactin-V4 | 0.5527 | -0.0168 | -0.0285 | -0.0209 | -0.0183 | -0.0373 | -0.0731 | -0.022 |
| Lactin-V5 | 0.1476 | -0.1137 | 0.0029 | -0.0852 | -0.0772 | -0.0509 | 0.0397 | -0.022 |
