# Supplementary figures and images for: An in silico framework for the rational design of vaginal probiotic therapy
Source: PLoS Comput Biol. 2025 Feb 14;21(2):e1012064. doi: 10.1371/journal.pcbi.1012064 (PMC11867318; doi:10.1371/journal.pcbi.1012064)

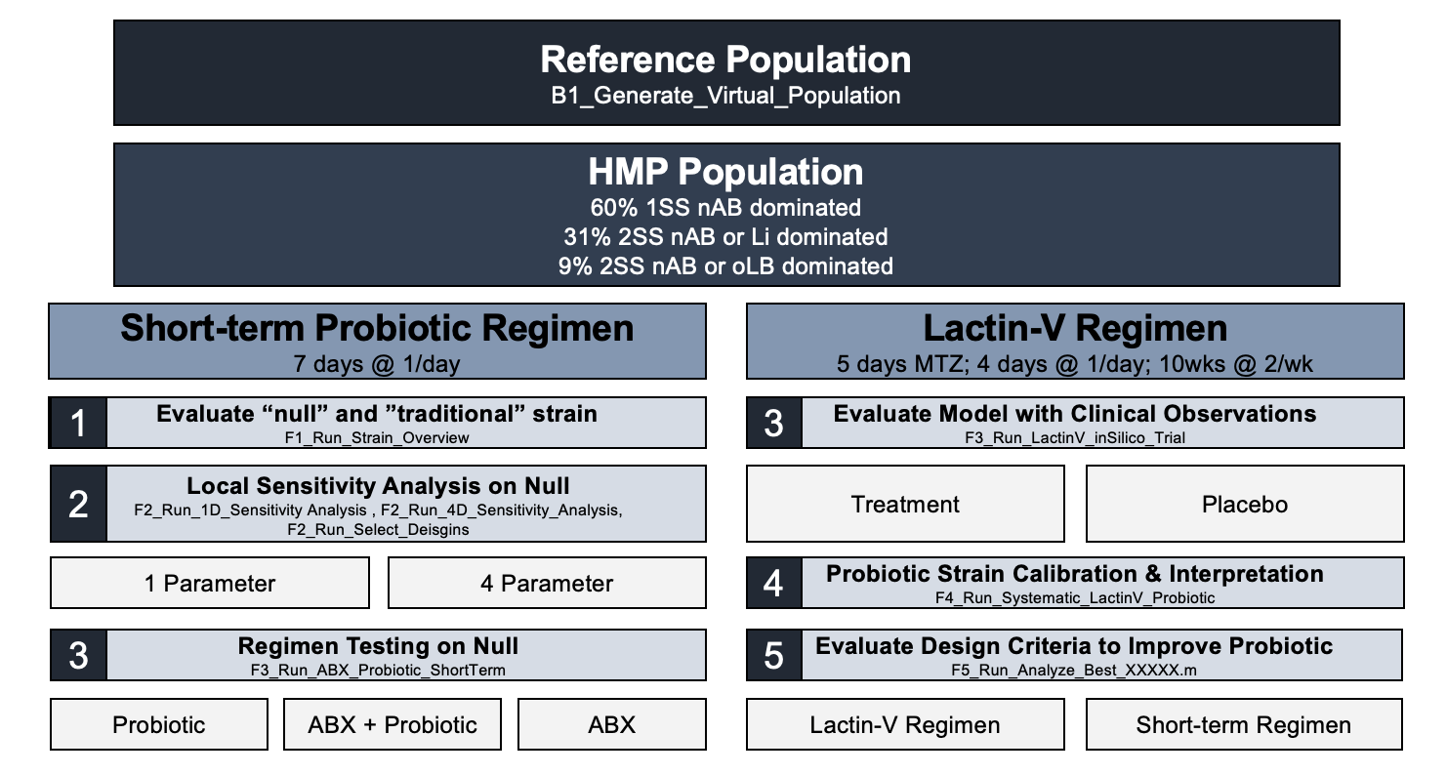

Supplement: S1 Fig — A virtual population was generated based on the Human Microbiome Project cohort. The analysis included two regimen types, a short-term probiotic regimen with no antibiotic pre-treatment and the regimen described in a phase 2b Lactin-V clinical trial (Cohen et al. 2020). In this framework, we tested various hypothetical probiotic strains, focusing on their interaction parameters with endogenous species. Additionally, we simulated our model using different probiotic and antibiotic regimens. Through these simulations, we identified the most important probiotic design criteria to address the issue of recurrent BV. (TIF) [file pcbi.1012064.s005.tif]

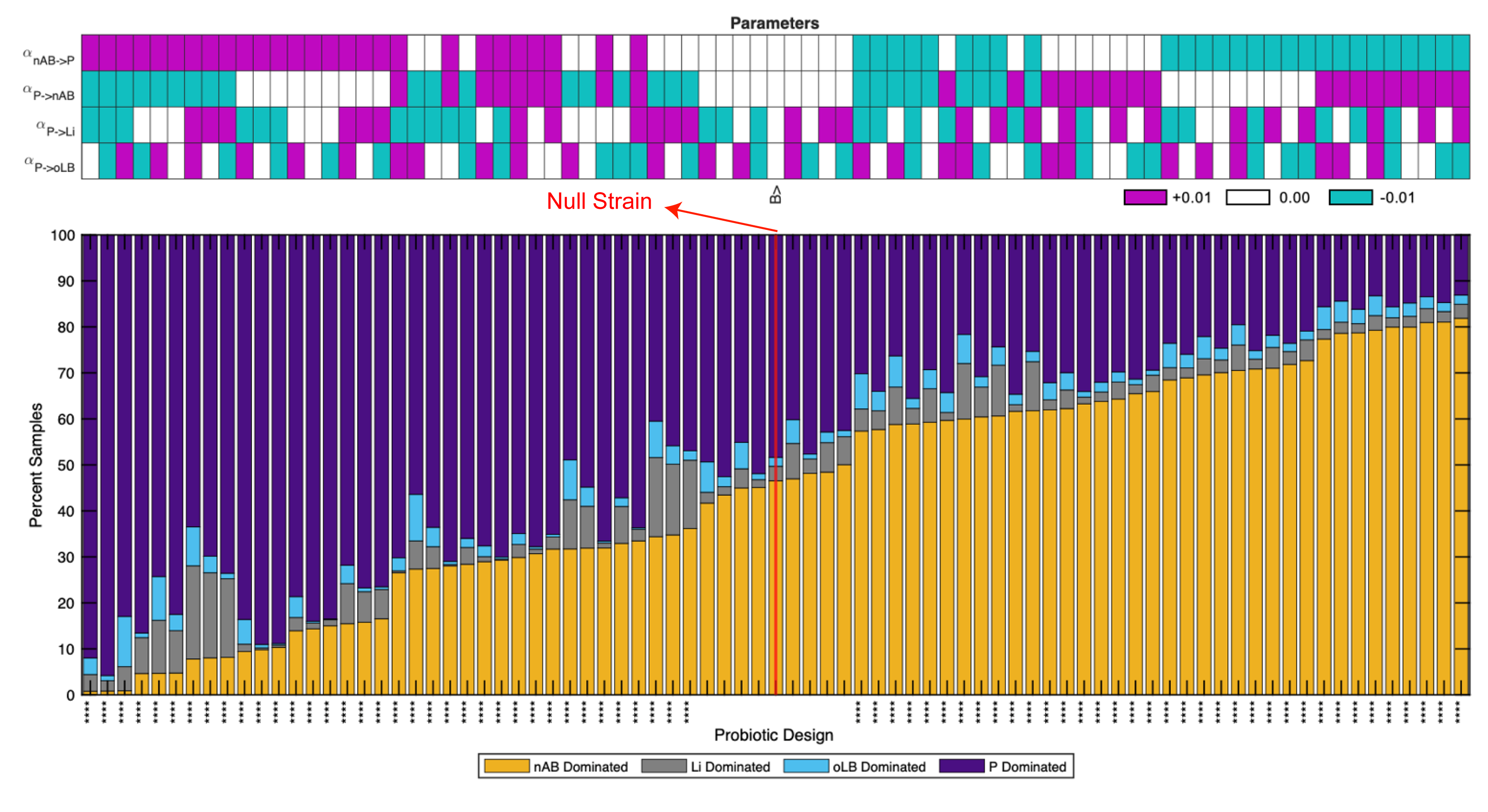

Supplement: S2 Fig — The top sensitive parameters for BV clearance and top parameters for modulating endogenous Lactobacillus spp. levels were modified systematically from -0.01, 0.00, +0.01 density-1d-1 from the null probiotic strain parameter values giving rise to 81 possible parameter combinations (top heatmap). The percent of the 2,000 in silico BV+ subjects that elicited a certain response type at 12months post therapy cessation were visualized (nAB-dominant, Li-dominant, oLB-dominant, or P-dominant). Data is plotted from most efficacious (left) to least (right). The null probiotic strain is indicated by “B>” and the red line. A chi-square test was performed to identify whether there is a significant difference in terms of efficacy between each of the 81 possible parameter combinations and the null strain. Asterisks indicate a significant change in efficacy relative to the null probiotic strain. (TIF) [file pcbi.1012064.s006.tif]

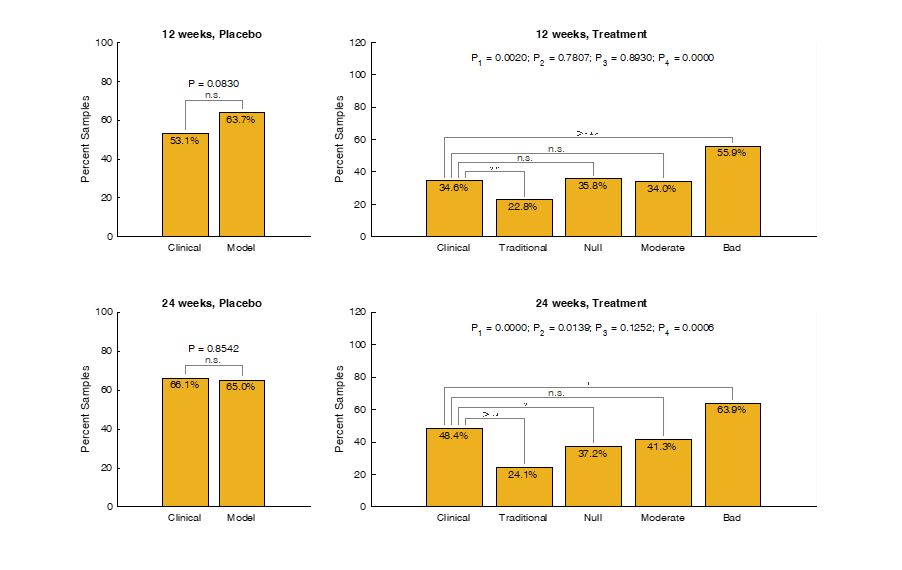

Supplement: S3 Fig — Comparison of model predictions with Lactin-V trial results at 12 and 24 weeks for the placebo and the treatment arm. For the treatment arm, 4 strains were simulated by the model encompassing a traditionally designed probiotic, null probiotic, moderately/conservatively designed probiotic, and bad/negative control probiotic. The impact of antibiotic was simulated at a magnitude (-3.82 d^-1) that was equivalent to the most sensitive G. vaginalis strain in Mayer et al. 2015 [69]. (TIF) [file pcbi.1012064.s007.tif]

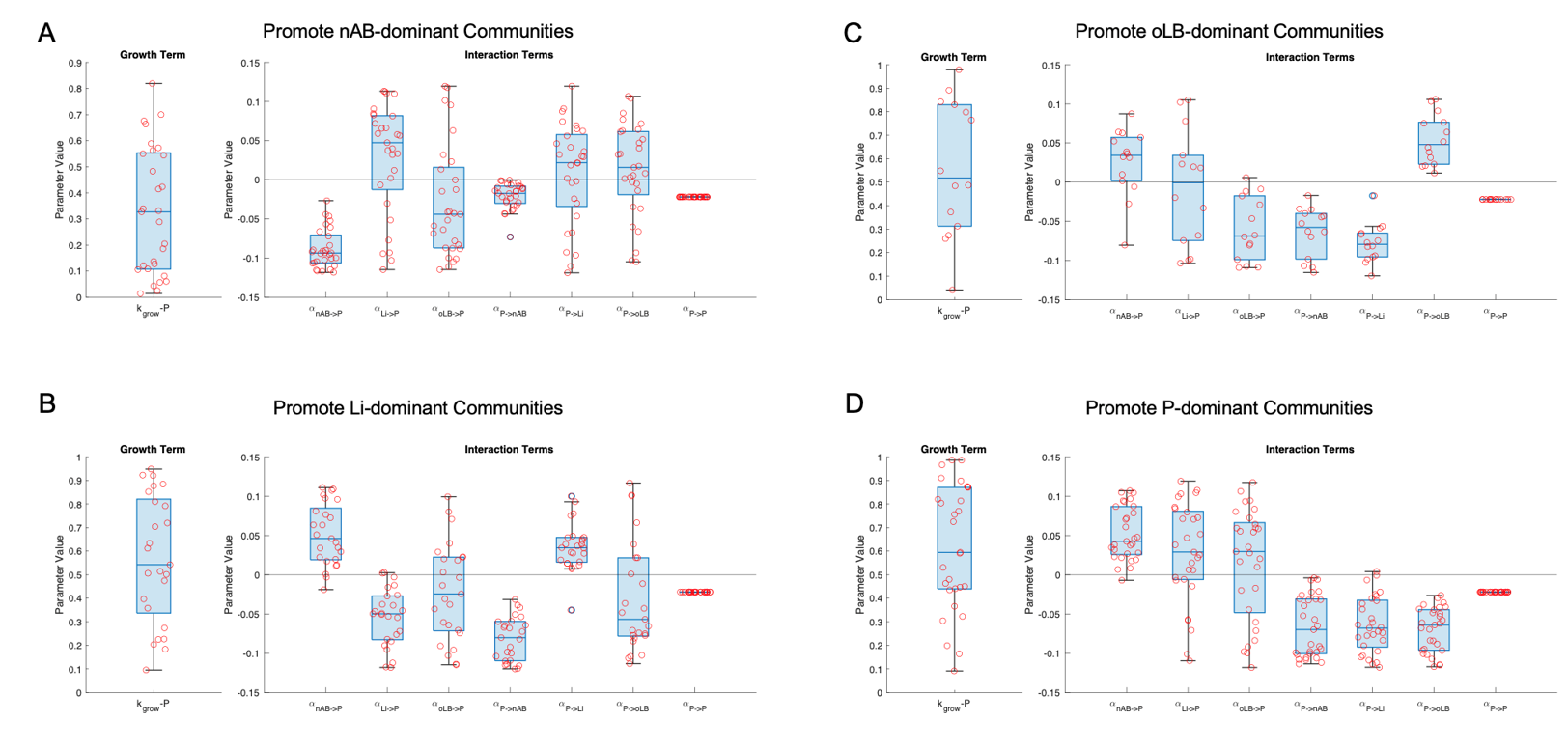

Supplement: S4 Fig — Strains in the 90th percentile or higher for imparting a certain compositional effect across the virtual population are visualized. (A) Consistently promote nAB-dominant communities (B) Li-dominant communities (C) oLB-dominant communities (D) P-dominant communities. (TIF) [file pcbi.1012064.s008.tif]

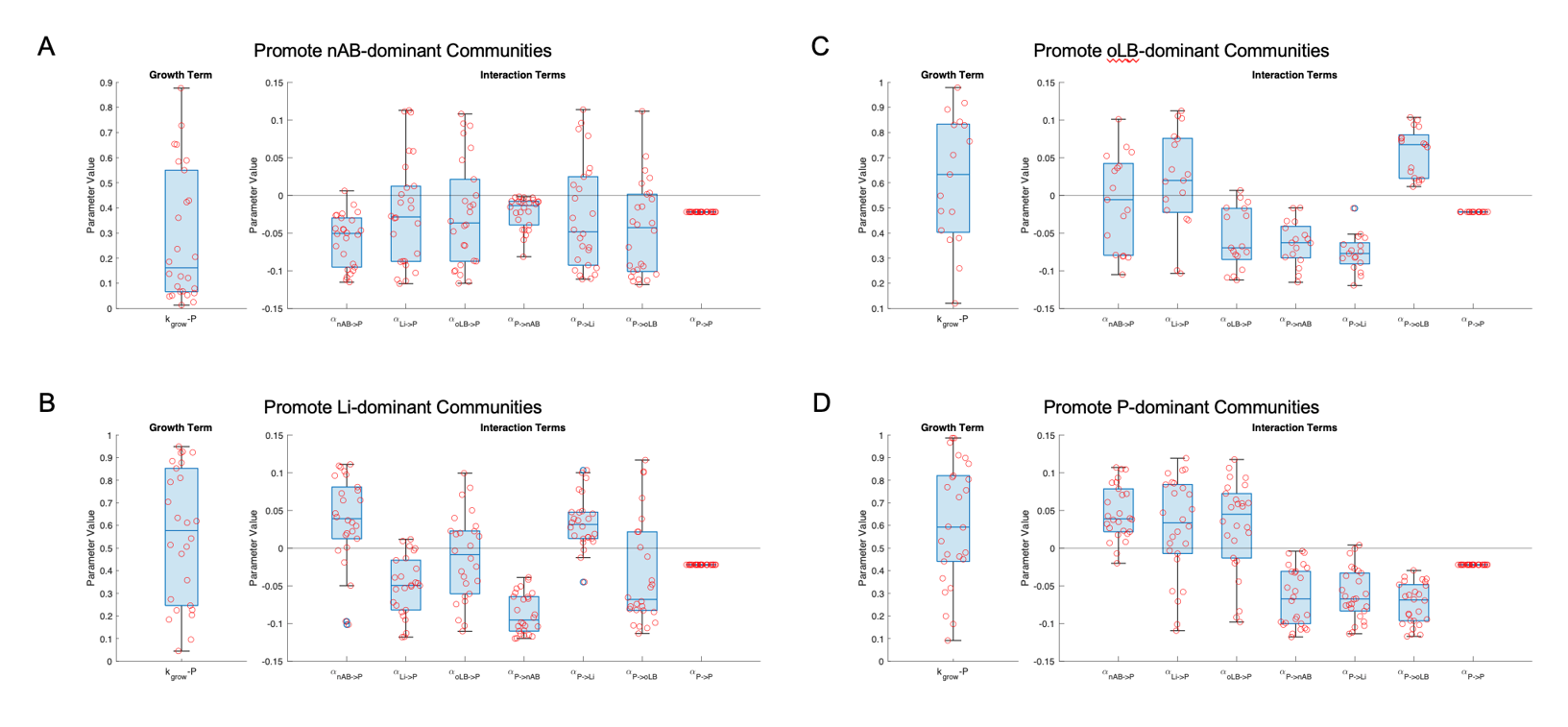

Supplement: S5 Fig — Strains in the 90th percentile or higher for imparting a certain compositional effect across the virtual population are visualized. (A) Consistently promote nAB-dominant communities (B) Li-dominant communities (C) oLB-dominant communities (D) P-dominant communities. (TIF) [file pcbi.1012064.s009.tif]

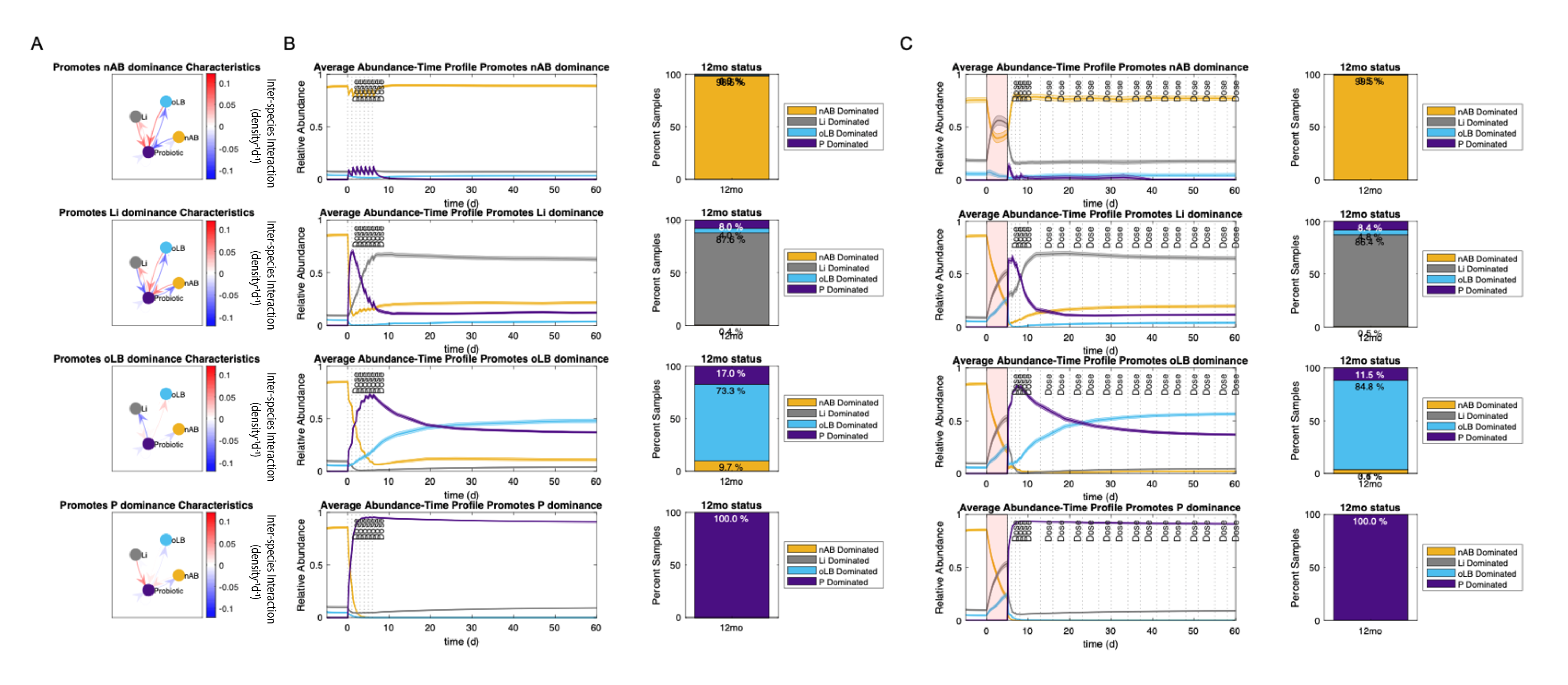

Supplement: S6 Fig — (A) Interspecies interaction parameter values of top probiotic strains for the short-term probiotic only regimen. (B) Abundance-time profile of community groups for each probiotic strain and predicted 12mo frequency of response types. (C) Lactin-V regimen abundance-time profile and predicted 12mo frequency of response types for strains defined in (A). Red indicates time of antibiotic dosing. (TIF) [file pcbi.1012064.s010.tif]
