# Supplementary material for: An in silico framework for the rational design of vaginal probiotic therapy
Source: PLoS Comput Biol. 2025 Feb 14;21(2):e1012064. doi: 10.1371/journal.pcbi.1012064 (PMC11867318; doi:10.1371/journal.pcbi.1012064)
Supplement: Attachment — Submitted filename: Reviewer Response.docx [file pcbi.1012064.s012.docx]

Reviewer's Responses to Questions

**Comments to the Authors:
Please note here if the review is uploaded as an attachment.**

**Reviewer #1:** The authors developed a comprehensive in silico framework to guide the rational design of more effective vaginal probiotic therapies. By simulating the complex microbial interactions in the vaginal ecosystem, the presented model can identify key parameters that govern the stability and resilience of different vaginal microbiome states.

Positive aspects:
- It provides a quantitative approach to understand vaginal microbiome dynamics
- It can help identify critical microbial interactions and parameters that influence microbiome stability
- It has the potential to guide the development of more targeted and effective probiotic interventions

Potential limitation/negative aspect:
- The model relies on simplifications and assumptions about the vaginal microbiome that may not fully capture its complexity

Main comments
My main concern centers on the authors' treatment of species proportions during evaluation. The focus seems solely on the dominant species, regardless of its proportion (e.g. 99% or 51%). This overlooks the case where the second most abundant species might be nearly as prevalent as the dominant one. Such close proportions between species don't seem to be considered in the analysis. This approach lacks clear justification in the paper, and the potential implications of this choice are left unexplored. I believe there's a need to refine this measurement methodology to better capture the species distribution and their potential impact. Having a diversity measure, such as an entropy measurement, could clarify the distribution at each time point.

We have now added clarification that the treatment of species proportions in this manuscript was not based on metrics chosen by the authors, but rather using metrics that are used broadly as the current clinical/experimental standard. We classified species response types from our models to be consistent with how the VMB has been historically classified in the clinic with community state types (CSTs)[1-4]. Unlike the gut microbiome, the vaginal microbiome is commonly associated with dominance of one species, particularly *Lactobacillus* spp. (CST -I, -II, -III, and V) or a polymicrobial community where no one species is dominant (CST-IV). Established classification methods for CSTs involve a nearest centroid classifier that converts relative abundance to a CST classification. Centroids for this classification are defined based on those reported in a landmark paper (France et al. 2020) with the VALENCIA framework [1].

While a major drawback of CSTs is the simplification of relative abundance to a categorical variable, it does reduce some statistical challenges associated with compositional data such as spurious correlations between species in the community [1]. Employing this method in our approach was essential for direct comparison to experimental and clinical assessments. This approach is also illustrated in our previously published work (Lee et al., Plos Comp Bio 2023, [2]). It is possible in future work we could explore the subtleties of communities that have slightly different compositions within CST, but for this foundational manuscript we believe it is important to stick to current standards for classifying VMB communities.

In order to better clarify these important points in the manuscript, we have added:

Introduction (page 3): *“Clinical observations support that the VMB is typically dominated by a single Lactobacillus spp. or exist in a polymicrobial state where no one species dominates the community [12,13]. These compositional states are referred to as community state types (CSTs) and are identified through an established nearest centroid classifier called VALENCIA [1]*.”

Results (page 5): *“The impact of the probiotic on community composition was defined using VALENCIA, a classification algorithm that is used clinically to assign CSTs at a set evaluation time point [1]. These classifications included nAB-dominant (probiotic failure rate), or dominance by Lactobacillus spp. (probiotic efficacy rate): Li-dominant, oLB-dominant, or Probiotic (P)-dominant.”*

Regarding the success associated with Li, oLB, or Probiotic dominance, the introduction clearly distinguishes Li as less beneficial to health. Given its differences, I don't think it's appropriate to consider Li dominant and oLB dominant as equally successful outcomes. The rationale behind this equivalence isn't adequately explained in the paper, I suggest mentioning this point at least in the discussion.

While L. iners is associated with more frequent transitions to BV, individuals with Li dominance are still considered healthy (e.g. do not have any adverse clinical symptoms). Thus, in regard to treatment outcomes we consider anything that would be associated with reduced symptoms to be a successful therapy at a set evaluated time point. We do agree that Li and oLB are not equally successful outcomes, though the model does enable the ability to distinguish between the two and suggests parameters for promoting dominance of Li vs. oLB. We have now added to the text to the manuscript better clarify this point:

Introduction (page 3): *“A fourth Lactobacillus sp., L. iners, is linked to CST -III and lacks the ability to produce compounds most associated with the inhibition of pathogens (H_2_O_2_, D-lactic acid). While L. iners is still considered healthy and does not promote adverse clinical symptoms, it has been associated with shifts to a non-optimal composition characterized by an overgrowth of facultative and obligate anaerobes known as bacterial vaginosis (BV).”*

Additionally, there are instances where the reasoning for selecting a specific evaluation time point for particular regime is unclear. For example, in Figure 5, two regimes are presented at different end states without clear justification for the choice of these specific time points. For a meaningful comparison, these regimes should be depicted at the same end state to ensure a fair evaluation.

We chose these different time points to reflect the time point a given probiotic regimen was evaluated at in each respective published study (Table S2). However, we agree that for direct comparisons, it is important to be consistent with the evaluation time point. For the results presented in Figure 5, we have performed an additional PLS-DA analysis of the short-term regimen at 24 weeks (6 months) to better match the Lactin-V evaluation time point. For the short-term treatment, steady-state was achieved before the 6-month mark. As a result, the outcomes for the short-term therapy were equivalent at the 6-month and 12-month time points (Figure 1C also demonstrates that the results of probiotic short-term therapy are stable after 1 month). We have modified Figure 5 as shown below:

**Fig 5.** **Drivers of population level composition alterations for a short-term regimen and Lactin-V regimen.** Partial least squares discriminant analysis (PLS-DA) models were constructed using strain characteristics to predict a population-level profile type (consistently nAB, Li, oLB, or P-dominant across the 2,000 subject virtual population) after (A) short-term regimen of probiotic (7 days of once daily probiotic treatment) and (B) Lactin-V regimen of probiotic (5 days MTZ treatment followed by 11 weeks of probiotic treatment). In each regimen, the scores plots indicate how the five hundred hypothetical strains generated by Latin Hypercube Sampling (LHS) are distributed according to their response types in the new component space. The loadings plots show how much each parameter contributes to the separation of clusters across the corresponding latent variables, highlighting the influence of each parameter on the response types.

In general, I think the paper would greatly benefit from more intuitions for the model parameters, i.e., what the inter-species interaction terms represent. This would give a clearer model understanding.

We have now added more information about model interaction terms in results on page 5:

*“Within this model, the inter-species interaction terms quantify the impact of one species on another. The sign of each term or whether the interaction is not significant (zero value) can be related to ecological terms such as commensalism (+/0 or 0/+), symbiosis (+/+), amensalism (-/0 or 0/-), competition (-/-), or parasitism (+/- or -/+). For example, a commensal relationship is reported between nAB bacteria G. vaginalis and P. bivia due to the production of a metabolite that increases the growth of the other species (+/0). In contrast, a common example of an amensal or competitive interaction can be observed between Lactobacillus spp. which produce lactic acid or other inhibitory compounds against nAB while only some nAB may produce compounds like biogenic amines that inhibit Lactobacillus spp. growth (-/- or -/0 interactions).”*

Further detailed comments and suggestions are included in the attached PDF.

Thank you for these helpful corrections. We’ve corrected the typos and corrected the suggestions listed in the PDF that was attached.

**Reviewer #2:** Here the authors used model prediction to assess probiotic therapies from a phase 2b trial with Lactin V in the treatment of bacterial vaginosis. Overall the authors found that antibiotic therapy and increasing dosage of probiotics may prevent BV recurrence. This is a comprehensive paper and incredibly important for this highly understudied field of women’s health. Minor comments below.

1. The abstract could be more clear that this is a modeling/simulation driven paper and what is being used.

We have now modified the abstract to emphasize that that is a modeling/simulation-driven paper and that results are computational predictions.

2. The first paragraph of the results is unclear how the simulation actually is representative of “real life” scenarios, please clarify of possibly make a cartoon figure to make this more clear to the reader. Figure 1B attempts this but the “virtual patient” concept is confusing to this biologist.

The virtual patient concept is based on a recently published paper (Lee at al., Plos Comp Bio 2023), where we created virtual patient populations by sampling parameters ranges that represented different cohorts. In this manuscript we use 2000 BV+ virtual patients from the HMP cohort that were generated in the previously published work. To clarify this concept for the current manuscript we have:

We have clarified this in the Results on page 5: *“In order to capture heterogeneity observed in microbial communities we used virtual patient populations generated in a previously published paper (Lee et al., 2023) [42]. Briefly, this methodology generated base virtual population using Latin Hypercube Sampling of defined parameter distributions from literature (30,000 parameter sets; S1 Table, S1 Fig). From the base virtual population of Latin Hypercube Sampling- generated parameter sets, 2,000 virtual patients were selected to replicate a distribution of BV-relevant CST equilibrium behaviors observed in the HMP cohort clinically [42]. This population was used to test different probiotic characteristics and regimens. BV-relevant CST equilibrium behaviors were defined as virtual patients that were analytically predicted to be nAB- dominant at steady-state.”*

We have edited the caption of Figure 1 to reference Lee et al. [42], the previously published work where a virtual HMP BV+ patient population was generated.

We have also edited the Methods on page 18: *“To test the impact of a probiotic strain at the population level, a virtual patient population was generated using Latin Hypercube Sampling of physiologically defined parameter ranges as described in Lee et al. 2023 [42] (S1 Table). Briefly, a large collection of virtual patients was generated (30,000) and then resampled to match clinically observed CST equilibrium behavior distribution pattern of the Human Microbiome Project Cohort (HMP) described in Lee et al. 2023. The CST equilibrium behavior describes the stability in CST classification over time, where subjects that consistently exhibit the same CST are considered mono-stable (1SS) and those that switch between different CSTs are considered multi-stable (2SS). Model predictions were related to these CST equilibrium behaviors using a nearest centroid classifier of the predicted relative abundances, and whether the system exhibited mono or multiple equilibrium states referred to as CST equilibrium behavior. The centroids for the nearest centroid classifier were determined from VALENCIA as described in Lee et al. 2023 [14,42]. Probiotic strains were tested on the subset of 2,000 HMP cohort virtual patients that could obtain a nAB-dominant (BV positive) state at equilibrium which includes the 1SS nAB-dominant (60%), 2SS nAB-dominant / Li-dominant (31%), and 2SS nAB-dominant / oLB-dominant CST (9%) equilibrium behaviors. The HMP cohort had a similar frequency of self-identified White/Caucasian subjects relative to persons of color as the Lactin-V cohort (32% versus 35%, respectively) [43].”*

3. In figure 3, the stats *** make the figure very busy. Perhaps make a small table of statistical comparisons instead.

All comparisons in this figure were significant. To improve the figure, we have now removed the *** from Figure 3 and indicated significance in the legend. We have replaced it with the new figure provided below:

**Fig 3. Selection of Optimal Probiotics**. Based on the local sensitivity analysis in Fig 2, hypothetic probiotic strains were selected with 1, 2 and 3 parameter changes to observe how much impact the combination of parameters could have on treatment outcomes. The parameters that were varied for each hypothetic strain are shown in the heatmap blow the plots. The heatmap below the plots indicates the parameter change and the value of the change: no change (white), +0.01 (pink), -0.01 (green). (A) Hypothetical strains that would be predicted to improve probiotic efficacy by reducing nAB-dominant response types. (B) Probiotics that boost endogenous oLB populations to increase oLB-dominant response types. Plots indicate the percentage of the 2,000 in silico BV+ samples for a given response type. Statistical comparisons were made with chi-square tests and all responses were evaluated at 12 months post treatment cessation. All comparisons were significantly different (p<0.001).

4. The figure legends should indicate if the data is based on virtual vs real patients

All figures with the exception of Figure 4 are based on simulations of virtual patients. We have now modified the captions of Figures 1-7 to better reflect this.

**Reviewer #3**: Authors develop a computational framework to assess the efficacy of vaginal probiotic treatment regimens to combat Bacterial vaginosis. They develop a mechanistic model based on gLV dynamics and incorporate the impact of antibiotic use in combination with the impact of species competition to understand the community composition after the application of various treatment regimens. I appreciate the work the authors put it, and I fundamentally believe in their approach in supporting research in probiotic design. However, the manuscript in its current form is confusing to the reader.

One of my major concerns is the ambiguity around methods, especially about the choice of parameter distributions and how the model is calibrated to recapitulate the results of the clinical trials and the antibiotic treatment.

Seems like the parameter ranges are derived from the previous publications in the literature (Table S1), but the steady state community composition is "related" to the clinical data using a nearest centroid classifier?

We appreciate these comments, which are similar to those made by Reviewer 1 above. As described above, we classified steady state community compositions in model predictions to be consistent with how the VMB has been historically classified in the clinic with community state types (CSTs)[1-4]. These established classification methods for CSTs involve a nearest centroid classifier that converts relative abundance to a CST classification. Centroids for this classification are defined based on those reported in France et al. 2020 with the VALENCIA framework [1] which is broadly used in VMB research. The virtual population is composed of many of these virtual patients where have been selected based on the frequency at which each CST is observed in the Human Microbiome Project (HMP) cohort and appears in our previously published work [2,5].

We have now clarified this in the Introduction (page 3) and the Results (page 5) as listed above in the response to Reviewer 1.

Another major concern is the lack of quantitative support for the interpretation of the results. Authors use adjectives like "high/low" degree of sensitivity without proper methods to support these comparisons.

To better quantify parameter sensitivity metrics, we have now employed a previously published method that quantifies the change in model outcome as a function of the change in each parameter value [16]. We used this analysis for each parameter according to four possible response types.

A summary of the method is below:

Using the equation (ΔY/ΔP)×(P0/Y0):

- ΔY is the difference between the fraction of virtual patients in each response type at the minimum and maximum parameter values tested, as shown in Figure 2.
- ΔP is the difference between the minimum and maximum parameter values.
- P0 is the initial value of the parameter.
- Y0 is the outcome based on P0.

For growth rate and self-interaction parameters, P0≠0. However, for inter-species interaction parameters, P0=0, similar to the null strain. Consequently, for inter-species calculations, we used P0=0.01 and Y0 as the output where P0=0.01.

For instance, for αnAB→P:

- ΔY=Y(0.1)−Y(−0.1)=0−0.93,
- ΔP=0.1−(−0.1)=0.2,
- P0=0.01,
- Y0=Y(0.01)=0.14.

We repeated this analysis for each response type, and the results are summarized in the below and we have created a new table provided as Supplementary Table S3.

Our main analysis to identify the most sensitive parameters is based on the failure response, which is predominantly determined by nAB. Among the interaction parameters, the most sensitive parameter is identified as α_nAB→P_, with a metric value of -0.33. Furthermore, we found that the most sensitive parameter for the oLB dominant response is also α_nAB→P_.

|  | nAB dominant |  | Li dominant |  | oLB dominant |  | P dominant |
| --- | --- | --- | --- | --- | --- | --- | --- |
| Parameter | Metric Value |  | Metric Value |  | Metric Value |  | Metric Value |
| α_nAB->P_ | -0.326 |  | -0.13 |  | -0.35 |  | 0.059 |
| α_P->nAB_ | 0.072 |  | -1.028 |  | -0.181 |  | -0.066 |
| α_P->Li_ | 0.021 |  | 0.248 |  | -0.04 |  | -0.059 |
| α_Li->P_ | -0.038 |  | -0.089 |  | -0.031 |  | 0.035 |
| α_P->oLB_ | -0.017 |  | 0.158 |  | 0.302 |  | -0.036 |
| α_oLB->P_ | -0.014 |  | -0.009 |  | -0.158 |  | 0.016 |
| k_grow-P_ | -0.781 |  | -0.018 |  | -0.025 |  | 0.824 |
| α_P->P_ | 0.854 |  | 0.042 |  | 0.049 |  | -0.945 |

We believe the interpretation of the self-interaction term might be somewhat misleading because it dictates the abundance of the probiotic (carrying capacity). Intuitively, if you increase the ability of the probiotic to grow to higher abundances, it will naturally become more dominant than other species in the community. This is likely influenced by the culture media or vaginal microenvironment, which is not something that can easily be replicated or selected for in vitro.

To better clarify in the manuscript, we have added the following paragraph on page 8:

*“To determine which parameters are most associated with improving probiotic efficacy (frequency of Li-, oLB-, P-dominant states post-treatment), local sensitivity analyses were conducted for all parameters describing probiotic strain characteristics. The level of sensitivity for each parameter and response type, was quantified using a previously published method, which normalizes the changes in outcome by the change in parameter value [44] (Fig 2). Notably, among the interaction parameters, the most sensitive parameters for the nAB dominant response (probiotic failure) were between nAB and probiotic (α_nAB→P_ and α_P→nAB_) with the highest absolute scores (-0.32 and 0.07, respectively; Table S3).”*

There is also a general lack of citations/references in the main text and figures captions are not sufficient. Please find my detailed comments below.

Abstract
"Bacterial vaginosis is a common condition characterized by a … ". Use the abbreviation BV here, as it appears in the rest of the abstract.

We have modified the abstract to specify that we abbreviate bacterial vaginosis as “BV”.

Introduction
References for the community state types? CSTs, CST -I, CST -II, CST -V are not very clear to the reader who is not very familiar with the VMB.

This is an important point that was also noted above by Reviewer 1. We classified species response types from our models in a similar manner that has been applied clinically, which is by using a nearest centroid classifier (VALENCIA) [1]. Using this standard method was essential to directly parallel clinical analysis and assessment. Clinical studies indicate that vaginal microbiome communities exist in community state types (CSTs) based on *Lactobacillus* spp. which tend to dominate a community or a community where no one species is dominant (associated with BV). Historically CSTs were characterized through clustering methods, but more recent approaches with improved reproducibility use a nearest centroid classifier. To mirror the methodology of VALENCIA, we used a similar approach where we used the VALENCIA centroids reported for each CST in our methodology [3]. We have updated the text on page 3 to better clarify the concept of CSTs and provided references (12,14 in the text), and also *included below:*

*“Clinical observations support that the VMB is typically dominated by a single Lactobacillus spp. or exist in a polymicrobial state where no one species dominates the community [12,13]. These compositional states are referred to as community state types (CSTs) and are identified through clustering methods or more recently through a nearest centroid classifier called VALENCIA [14].”*

The following references were added:

VALENCIA: France MT, Ma B, Gajer P, Brown S, Humphrys MS, Holm JB, et al. VALENCIA: a nearest centroid classification method for vaginal microbial communities based on composition. Microbiome. 2020;8: 166. doi:10.1186/s40168-020-00934-6

CSTs: Ravel J, Gajer P, Abdo Z, Schneider GM, Koenig SSK, McCulle SL, et al. Vaginal microbiome of reproductive-age women. PNAS. 2011;108: 4680–4687. doi:10.1073/pnas.1002611107

L. iners is "less" associated with health and commonly associated with BV? This sentence should be more clear, and needs a reference.

This is an important point which was also noted above by Reviewer 1. As described above, we have added the following to the text to clarify this point:

Introduction (page 3): *“A fourth Lactobacillus sp., L. iners, is linked to CST -III and lacks the ability to produce compounds most associated with the inhibition of pathogens (H_2_O_2_, lactic acid)[17,20]. While L. iners is still considered healthy and does not promote adverse clinical symptoms, it can be more associated with shifts to a non-optimal composition characterized by an overgrowth of facultative and obligate anaerobes known as bacterial vaginosis (BV)[1,17,21].”*

New references that have been added to the manuscript are:

Witkin SS, Mendes-Soares H, Linhares IMJayaram A, Ledger WJ, Forney LJ. Influence of Vaginal Bacteria and d- and l-Lactic Acid Isomers on Vaginal Extracellular Matrix Metalloproteinase Inducer: Implications for Protection against Upper Genital Tract Infections. mBio. 2019; 4. doi: 10.1128/mbio.00460-13

Amabebe, E., & Anumba, D. O. The Vaginal Microenvironment: The Physiologic Role of Lactobacilli. *Frontiers in Medicine*. 2018; *5*: 389042. [doi: 10.3389/fmed.2018.00181](https://doi.org/10.3389/fmed.2018.00181)

Edwards VL, Smith SB, McComb EJ, Tamarelle J, Ma B, Humphrys MS, et al. The Cervicovaginal Microbiota-Host Interaction Modulates Chlamydia trachomatis Infection. mBio. 2019;10. doi:10.1128/mBio.01548-19

The "Non-optimal" part of the nAB should be briefly defined in the introduction. Same for "optimal". What do these terms mean exactly? Pathobiont vs symbiotic commensal?

We have added some text in the introduction to help clarify what species are referred to when we state “non-optimal” in nAB on page 3:

*“Two species most commonly associated with BV are Gardnerella vaginalis and Prevotella spp., which can exist at low levels even in Lactobacillus spp. dominant communities [12]. However, typically no one species dominates the community in non-optimal conditions associated with BV and herein we generally refer to species that make up these communities as non-optimal bacteria (nAB) dominant”*

In simplest terms, non-optimal bacteria would fit in with a pathobiont definition; however, this term is not as commonly used for BV and the vaginal microbiome. This convention is due to the fact that some individuals can have a community that is not dominated by species considered to be optimal (Lactobacillus spp.) but still appear to be healthy or asymptomatic.

Results
Reference for the method "Latin Hypercube Sampling"? In general, I appreciate the references in the supplementary but the main text needs better references. I will not point them out after this point, please go through the main text again and provide references for scientific/clinical statements and methods.

We have gone through the text and added additional references such as Mckay et al. 1979 for Latin Hypercube Sampling [9]. We also added references for generalized Lotka-Volterra models (page 5), PLS-DA and Benjamini & Hochberg procedure (page 20) (reference numbers in the text are 35-38, and 50 and 70).

Right after the first paragraph of results, I still don't know what "probiotics" contain. Is it oLB? Is it any other Lactobacillus spp.? It makes it hard to interpret the CST classifications you defined to quantify the efficacy of the treatment.

Probiotics developed for the vaginal microbiome are most commonly *Lactobacillus* spp., but in some cases have been *Bifidobacterium or Bacillus* spp. *[11].* Table S2 has a compilation of a variety of different probiotics that have been evaluated in the literature for efficacy against BV.

In this platform, the probiotic species is representative of whatever probiotic species or strain that is to be evaluated. Here it assumes a single strain, and the parameters that are associated with its growth and interaction with endogenous *Lactobacillus* spp. and non-optimal bacteria such as *G. vaginalis* or *P. bivia.* For example, when simulating the Lactin-V regimen the probiotic strain would be representative of *L. crispatus* CTV-05 [10]. Therefore, the interpretation of the results would be that when the communities are probiotic-dominant, the probiotic is classified as being the most dominant species. *L. iners* or oLB dominant indicates endogenous *Lactobacillus* spp. rather than the probiotic.

We also simulate a “null strain” as a control comparison, which has no interactions with the surrounding community. In the manuscript we have now clarified this in the Results (page 6):

What is a null and what is a traditional probiotic strain? What do these groups contain in terms of species?

This is an important point that was also noted by other reviewers. To clarify these hypothetical groups, we have now modified the manuscript on page 6:

*“For simplicity, a hypothetical null probiotic strain and a traditional probiotic strain were created to explore the possible response types. The null probiotic strain (control strain which exhibits no impact on the endogenous community and vice versa) was modeled as having negligible interspecies interactions (α_nAB→P_, α_Li→P_, α_oLB→P_, α_P→nAB_, α_P→Li_, α_P→oLB_ = 0.0 density^-1^d^-1^) a moderate growth rate (k_grow-P_ = 0.5 d^-1^), and a moderate self-interaction term (α_P→P_ = -0.022 density^-1^d^-1^). The range of growth and self-interaction terms were characterized from in vitro experiments [37,72]. Resulting model predictions indicated that the null probiotic strain was predicted to have primarily P-dominant or nAB-dominant response types at each evaluation point, with a maximal failure rate of 46.5% at 12 months (Fig 1C). A second strain, designed as a proof of concept example with traditional considerations of ensuring the probiotic strain could inhibit nAB growth was simulated using the null probiotic strain parameters and α_P→nAB_ set to -0.01 density^-1^d^-1^ (Fig 1D).”*

How do you decide whether the growth rate of 0.5d-1 is moderate? Or self-interaction term of -0.022 density-1d-1? What is the rationale behind these quantitative cutoffs?

Previously, in Lee et al. 2023 [2] parameter ranges were determined for inter-species interaction terms and growth parameters. The interspecies interaction terms were calculated from *in vitro* co-culture experiments and the minimum and maximum strengths were used as the lower and upper bounds to sample from LHS [12].

Growth terms (growth rate and carrying capacity) were also characterized from *in vitro* experiments [13,14] to determine the spread of the parameters and *in vivo* estimates based on typical ranges from the gut microbiome [15] to capture upper and lower bounds.

To define a moderate parameters, we assumed a value that was approximately the middle of the upper and lower bounds. Growth rate is in the range of 0.1-1. As a result 0.5 can be considered as moderate. -0.022 is the average value of the range -0.004 to -0.04 for self-interaction parameters which are provided in S1 table.

To better clarify, this statement is added on page 6:
*“The range of growth and self-interaction terms were characterized from in vitro experiments. The growth rate ranges from 0.1 to 1, with 0.5 considered moderate. For the self-interaction parameters, the average value is -0.022, derived from the provided range of -0.004 to -0.04 in Table S1.”*

P-dominated or P-dominant? Pick one and stick with it for the entire text.

We have revised the manuscript to consistently use P-dominant.

'The traditional strain had a lower rate of treatment failure, with nAB dominant communities comprising 32% of virtual patients for times greater than 1 month post-treatment cessation (P < 1x10-6, χ2 test between null and traditional at each timepoint 1 month and greater). ' -> this sentence is a bit confusing. When are you sampling the microbiome composition post-treatment?

We agree this is confusing and have now modified it in the text. This section is referring to Figure 1C, which shows the frequency of each response type across the virtual population evaluated at treatment cessation, 1 week, 1 month, 3 months, 6 months, and 12 months post treatment cessation for the null strain and traditional strain. Performance at reducing the frequency of nAB dominant responses in the virtual population were compared between the null strain and traditional strain at each time point. Because steady-state was achieved around the 1 month time point, the statistical comparisons of all later time points resulted in the same frequency comparison and significance value which is what was reported in the text.

I assume the composition is dynamic, moving toward a new equilibrium after treatment cessation. This means depending on the time of sampling, you will see different percentages of nAB within the community composition. Similarly, with the chi-square test what are you comparing exactly? I assume it is the binary indicator of failure vs success since you are using chi-square?

Yes, the response is dynamic and we were comparing the binary indicator of success vs failure as defined by the frequency of nAB dominant responses in the 2,000 patient virtual population as the metric of the failure rate. Steady-state is reached around 1 month after treatment cessation. We did a chi-square test comparing null vs traditional strain at the same time point for each time point evaluated. Since the frequencies were relatively stable after one month, the results were similar. As referenced above, we have clarified this on page 7. We added the following statement:

*“Steady-state was achieved around 1 month for the proportion of nAB dominant response types after treatment where the traditional strain exhibited a higher frequency of treatment success defined by lower frequency of nAB dominant virtual patients after treatment compared to the null group. At and after the 1 month evaluation time point, 32% of virtual patients were nAB dominant for the traditional strain compared to 46.5% for the null strain, which was significantly different (P < 1E-6,* ***χ^2^-****test for frequency of nAB dominant responses).”*

In general, I am a bit confused about the null strain. You put a species that is not interacting with any other species, but it is still growing. So it has a niche that is not occupied by all the rest of the microbiome?

Yes, that is correct. We wanted something that would be “inert” or have a null effect on the endogenous community that we could then use as a reference for the performance of varying probiotic strain characteristics (interactions with the endogenous community or growth characteristics). While this may not be the most realistic in the sense of what may actually occur for a typical probiotic strain, it provides a baseline/reference for what could positively or negatively impact the simulated probiotic strain’s performance. We have clarified this point in the results section on page 5, we have added the terms "control strain" and "hypothetical" to better clarify the role of the null strain.

I understand that you are trying to create an analogy to the "null" hypothesis here, but what you are trying to show is anyway about the "degree" of P-dominancy given how much P can inhibit the growth of nAB, no?

If so, why don't you play with that parameter on a continuous scale and plot 12mo P dominated percentage vs alpha_P->nAB? This would be much better to understand how much inhibition one needs based on the target community composition at 12 mo.

To clarify, in Fig 2, we performed a sensitivity analysis to identify how changes in probiotic parameters influence nAB dominance. In other words, we demonstrated the extent to which variations in the 8 probiotic parameters can inhibit the growth of nAB. In Figure 2 the relationship between all four response types are compared to changes in each probiotic strain parameter on a continuous scale (20 values in addition to the null value are used from the minimum and maximum range defined for each parameter).

The model was designed to measure the response type using specified parameter values. While plotting on a continuous scale could be another way to present our sensitivity results, we chose to use stacked bar charts to visualize all four possible response types. Additionally, we focused on using the nAB dominant response as the output because we considered Li, oLB, and P as optimal results in all of our simulations. Therefore, it was not applicable to plot only the percentage of P dominant against parameter values.

After reading the comments below, we have tried to better frame the purpose of figure 1 as a proof of concept of how our methodology works so that the reader is set up to understand the results of figure 2 which has the local sensitivity analysis of each probiotic strain parameter as on page 7:

*“A second strain, designed as a proof of concept example with traditional considerations of ensuring the probiotic strain could inhibit nAB growth was simulated using the null probiotic strain parameters and α_P→nAB_ set to -0.01 density^-1^d^-1^ (Fig 1D).”*

(As I continue reading, I started to understand that P compartment is "hypothetical", and does not contain any particular species yet, am I correct? This should really be clear to the reader at the beginning).

Yes, all the designed probiotic strains are hypothetical and created for simulation purposes. To make this clearer, we have added the term "hypothetical" in the Results section on page 7 and also in the abstract.

As I move, I see now my comment above is addressed in the parameter sensitivity analysis. I still keep it because this is how the reader thinks before they move on with the manuscript :) Given that the manuscript focuses on the interaction terms so much in the abstract and the introduction, I think you don't really need the null strain. You have all the information in the sensitivity analysis anyway.

We do think that null strain is important in that it serves as the control group, and it is hypothesized to have no interaction with host species. Clearly defining the null strain is important to better address the other types of hypothesized strains we created. For instance, in Figs. 3 and 4, we demonstrated how probiotic efficacy changes when using different probiotic strains, including the null strain. We compared how the failure rate varied with different strains. Moreover, in Fig. 3, one of our main comparisons was between the null strain and the strain named 2p: Counter, which had a significantly higher failure rate compared to the null strain. We have tried to better clarify the purpose of the null strain in the Results and Methods, as described above.

You mention the inhibitory compounds (D/L-lactic acid, hydrogen peroxide, or bacteriocins) during the results section, but in the introduction there is not much about the efficacy of these products, rather you only mention that L. iners is not capable of producing them. There is really no literature around the use of probiotics that produce these metabolites?

Thanks for this helpful point. We have incorporated more detail in the introduction on page 3 which includes:

*“These species are able to facilitate the acidification of the vaginal microenvironment through the production of D, L-lactic acid and are also reported to produce hydrogen peroxide, which are linked with decreasing susceptibility to sexually transmitted infections and inhibiting the growth of pathogens and other non-optimal species [1,15-18]. Additionally, Lactobacillus spp. can produce bacteriocins and surfactants which can also prevent colonization of non-optimal or pathogenic species. A fourth Lactobacillus sp., L. iners, is linked to CST -III and lacks the ability to produce compounds most associated with the inhibition of pathogens (H_2_O_2_, D lactic acid).”*

Regarding the interpretation of the local sensitivity analysis:

The "degree" of sensitivity is not quantified here, therefore you cannot use phrases like "more/most sensitive parameters" or "high degree of sensitivity" in the text. You need to quantify this first. You can employ something like random forests and predictor importance analysis given you have a high dimensional model and options for both a binary outcome of treatment failure/success or the degree of nAB domination.

This question is also answered above in this reviewer response file. We have now incorporated a sensitivity metric similar to the one described in Kraeutler et al. 2010 for local sensitivity analyses [16]. This metric is S_ij_ = (ΔY_i_/ΔP_j_)(P_o,j_/Y_o,i_), where i is the ith parameter and j is the jth response metric. Y indicates the response value for the parameter change, Y_0_ refers to the value with no change to the parameter (null strain). P0 is the initial value of the parameter. Y0 is the outcome based on P0. For growth rate and self-interaction parameters, P0≠0. However, for inter-species interaction parameters, P0=0, similar to the null strain. Consequently, for inter-species calculations, we used P0=0.01 and Y0 as the output where P0=0.01.

We repeated this analysis for each response type, and the results are summarized in the table provided on page 7 of this file. This table is also included in the supplementary file (Table S3). To make it more clarified for the readers, we have added the following statements on page 8 of the manuscript:

*“The level of sensitivity for each parameter and response type, was quantified using a previously published method, which normalizes the changes in outcome by the change in parameter value [40] (Fig 2). Notably, among the interaction parameters, the most sensitive parameters for the nAB dominant response (probiotic failure) were between nAB and probiotic (α_nAB→P_ and α_P→nAB_) with the highest absolute scores (-0.32 and 0.07, respectively; S3 Table).”*

"The importance of probiotic inhibition of nAB (nABP) is unsurprising, given the selection of probiotics that produce inhibitory compounds (D/L-lactic acid, hydrogen peroxide, or bacteriocins) for nAB is a standard practice [26–30]. In contrast, the high degree of sensitivity for nAB on probiotic (nABP) is less intuitive and not well characterized in vitro or in vivo." -> there is a typo here. You use the same interaction term for probiotic inhibition of nAB and nAB on probiotic. This is super confusing.

Thank you! There was a typo here and we fixed it and updated Figure 1.

Figure 2 needs a better figure caption. When is the time of sampling here? 12mo? (same goes for Fig. S2).

That’s an important point. These measurements are taken at 12months post-therapy cessation, and we have now clarified this in both figure captions.

Figure S2: All treatments are significantly better than the null strain with three stars? How is the efficacy compared here? This is the supplementary, you can afford to explain more in the figure caption and make these figures standalone.

In Fig. S2, stars indicate that the strain had a significantly different failure rate compared to the null strain (either better or worse). We performed a chi-square test to compare the failure rate of each of the 81 possible parameter combinations with that of the null strain (represented by the red line). To clarify how we compared the efficacy, we have added the following statement to the Fig. S2 caption:

*"A chi-square test was performed to identify whether there is a significant difference in terms of efficacy between each of the 81 possible parameter combinations and the null strain."*

What is a "select" strain? Are these "real" species or are these hypothetical parameter combinations? It's very confusing!

We have clarified here and throughout the text that the strains test are hypothetical. To fully address this and provide a better explanation for Fig. 3, we have added the following statement on page 8:

*"Based on the results of the sensitivity analysis, we aimed to identify the strain with the lowest failure rate. We simulated our model with three hypothesized strains, including the best strain given only one parameter is altered: 1-p traditional strain (positive α_nAB→P_), the best strain given two parameters are altered: 2-p best strain (positive α_nAB→P_ and negative α_P→nAB_), and when three parameters are altered the 3-p best strain (positive α_nAB→P_, negative α_P→nAB_, and positive K_grow-P_)."*

"All selected strains were significantly from the null strain."? Significantly what?

That was a typo. We fixed it by adding “different”. Statement has been modified: “All selected strains were significantly different from the null strain.”

Define MTZ again in the results before using the abbreviation.

The definition of metronidazole is now defined before MTZ on page 8.

Where does Lactin-V probiotic fall within all these ranges of hypothetical probiotics you have simulated so far in the manuscript?

Characteristics for Lactin-V, such a growth rate and interaction terms were not able to be identified from the literature. Thus, we benchmarked the performance of a few different hypothetical strains against the observed clinical performance of Lactin-V in Figure 4C. A transitional sentence was added to make this methodology more clear.

We have now clarified this in the text on page 11.

1. The “traditional” probiotic strain with strong inhibition of P on nAB (simulation positive control, -0.012 d^-1^density^-1^ α_P→nAB_).
2. The “null” probiotic strain.
3. A “moderate” strain with strong competition with endogenous *Lactobacillus* spp. vaginal microbiota (3X strength of α_P→nAB_), weak inhibition of nAB on P (1/2X α_P→nAB_), and strong inhibition of P on nAB (-0.012 d^-1^density^-1^ α_P→nAB_).
4. A “bad” strain with strong competition with endogenous *Lactobacillus* spp. (3X strength of α_nAB→P_), weak inhibition of P on nAB (1/2X traditional strain strength of α_P->nAB_), and strong antagonism of nAB against the probiotic (1X traditional strain strength of α_P→nAB_) (simulation negative control or bad strain).

We have also added the following text on page 11:

*“Altogether, these results suggest the Lactin-V probiotic strain (L. Crispatus CTV-05) has a a competitive interaction with endogenous Lactobacillus spp. and the probiotic has a stronger impact on nAB (α_P→nAB_) than nAB has on the probiotic (α_nAB→P_) similar to our hypothesized moderate strain.”*

Since the manuscript focuses a lot on the interaction parameters' influence on the outcomes, the section "Combinatorial regimens can lower BV recurrence rates" should provide some quantitative idea of what those parameters are for the Lactin-V probiotic treatment.

As described above, the Lactin-V interaction parameters are unknown, but we used the model to try to infer what they may be. We have clarified the text as described above.

Typo: "The simulation of the Lactin-V treatment arm included *for* possible probiotic strains"

This statement is removed and replaced by the following statement on page 10:

*“Since characteristics of Lactin-V strain, L. crispatus CTV-05 are not well characterized in the literature, four different hypothetical strains were benchmarked against the clinical results in the phase 2b clinical trial.”*

I am really lost about the simulations for the Lactin-V regimen. Isn't it one strain, Lactobacillus crispatus CTV-05? Why do you simulate multiple strains?

Correct, *L. crispatus* CTV-05 is one strain; however, we were not able to parameterize the strain based on information that is available in the literature. Therefore, we benchmarked some possible scenarios that could potentially replicate the performance of Lactin-V. We have clarified that in the text on page 9:

*“Since characteristics of Lactin-V strain, L. crispatus CTV-05 are not well characterized in the literature, four different hypothetical strains were benchmarked against the clinical results in the phase 2b clinical trial.”*

Sorry if I missed this, but the quantitative impact of ABX (the killing rate) on nAB, Li, oLB, and P is stated exactly where in the manuscript? It is hard to interpret the results of Figure 4 without that information.

We have added a description to the captions Fig S3, and Fig 4. In Fig S3 caption , the decay rate of ABX on nAB is (-3.82 d^-1) which is equivalent to the most sensitive G. vaginalis to ABX therapy reported by Mayer et al. 2015 [17]. In Fig 4, the decay rate is -2.64d-1 which is the average decay rate ranged from -0.95 d-1 to -5.44 d-1 reported by Mayer et al. 2015. Both captions are updated now to provide better understanding of ABX impact on nAB. Also, we assumed ABX doesn’t have any interaction with Li or oLB as *Lactobacillus* spp. typically resistant to metronidazole [18].

Figure caption of Fig. 5 is not sufficient. Also, what are "scores" and "loadings" on the y axis? Loadings mean eigenvalues? I see that this is in the statistical analysis, but the reader shouldn't have to go there to read Fig. 5. In general, please make all the figures standalone. The caption must be sufficient to understand and interpret the figure.

In a PLS-DA plot, "scores" and "loadings" have specific meanings:

1. **Scores:** Scores represent the coordinates of the original samples in the new component space defined by PLS-DA. They indicate the projection of the original data onto the latent components that the PLS-DA model has identified. Essentially, scores show how the samples are distributed in the newly created component space, which helps in visualizing the separation between different groups.
2. **Loadings:**  Loadings are scaled versions of eigen vectors by the square root of the corresponding eigen value, therefore the higher the value indicates higher contribution of the associated parameter contributes to the corresponding latent components. They are the coefficients that relate the original variables to the new latent components. Loadings help understand which variables are influential in defining each component and contribute to the separation between groups.

We used PLS-DA visualization to find the association between probiotic characteristics and response types. On the scores plot across x-axis (LV1) we see the separation of nAB from optimal species. Also, across y-axis we see a separation between Li and oLB. Then we can consider the loadings plot to find which features associated with which group. We made the Fig 5 caption more complete by adding the following text:

*“In each regimen, the scores plot indicates how the five hundred hypothetical strains generated by Latin Hypercube Sampling (LHS) are distributed according to their response types in the new component space. The loadings plots show how much each parameter contributes to the separation of clusters across the corresponding latent variables, highlighting the influence of each parameter on the response types.”*

Fig. 6 is hard to read in general. One really needs to zoom in.

We have clarified the plot for better visualization. Additionally, we used the hypothetical strains listed below for Figures 6 and 7. This table (Table S4) is also included in the supplementary materials.

|  | k_grow-P_ | a_nAB->P_ | a_Li->P_ | a_oLB->P_ | a_P->nAB_ | a_P->Li_ | a_P->oLB_ | a_P->P_ |
| --- | --- | --- | --- | --- | --- | --- | --- | --- |
| Top nAB | 0.2049 | -0.0465 | 0.11 | -0.0001 | -0.0083 | -0.0963 | -0.1047 | -0.022 |
| Top Li | 0.9489 | 0.1018 | -0.1176 | 0.0994 | -0.0986 | 0.0752 | -0.1042 | -0.022 |
| Top oLB | 0.7106 | 0.1009 | 0.0747 | -0.1124 | -0.0583 | -0.0827 | 0.0937 | -0.022 |
| Top P | 0.5305 | 0.0321 | 0.0994 | 0.0168 | -0.0523 | -0.0068 | -0.0381 | -0.022 |
| Best Fit Lactin-V | 0.253 | -0.101 | -0.0541 | -0.0509 | -0.0617 | -0.0601 | -0.0087 | -0.022 |
| Lactin-V2 | 0.8241 | -0.0174 | -0.1092 | -0.0573 | -0.0072 | -0.0613 | 0.0402 | -0.022 |
| Lactin-V3 | 0.3633 | -0.0148 | -0.0667 | -0.0902 | -0.1043 | -0.0135 | 0.0457 | -0.022 |
| Lactin-V4 | 0.5527 | -0.0168 | -0.0285 | -0.0209 | -0.0183 | -0.0373 | -0.0731 | -0.022 |
| Lactin-V5 | 0.1476 | -0.1137 | 0.0029 | -0.0852 | -0.0772 | -0.0509 | 0.0397 | -0.022 |

Discussion
Could you discuss why you have a statistically significant difference between the clinical results and your model in Fig. 4B at 12 weeks, despite having non-significant differences at 24 weeks? Whatever is different, it is transient in your model, which is a bit weird given that you give placebo. Since there is no perturbation to the system, it means there is something about the interaction/growth terms that lead to a similar steady state (24 weeks) but a transient difference in 12 weeks? If it were to be because of the interaction terms, then the steady state response should also be significantly different, right? Given that it is not, I wonder what creates this transient difference during the placebo.

The placebo arm still is given antibiotic therapy, which we have now clarified in the text and the figure 4 caption. We have added the following statement on page 10:

*“The placebo (5-day regimen of MTZ followed by the Lactin-V regimen with no probiotic strain simulated)”*

Thus, the difference of the placebo arm BV response rates at 12 weeks and 24 week is due to response to treatment with antibiotic which does not reach steady-state until after 12 weeks (6 months).

The reason for why the model is significantly different at 12 weeks and not significantly different at 24 weeks indicates that the model is less accurate at capturing the transient impact of antibiotic therapy (12 weeks), but performs better at predicting the long-term impact of antibiotic therapy (24 weeks). We added the following statement into discussion on page 16:

*“The model is more accurate at predicting the long-term impact of antibiotic therapy (24 weeks) than short-term (12 weeks), likely because it is less accurate at capturing the initial transient impact.”*

Clinical response to antibiotic therapy is variable as reflected in Mayer et al. 2015 [17]. For simplicity, we chose to use the average reported rate of nAB decline after treatment to simulate the antibiotic regimen in Figure 4 (2.64 d^-1^). We included an additional supplementary figure with a higher decay rate (stronger impact on decreasing nAB abundance) which better replicated the placebo regimen at 12 weeks (Fig S3).

We have referenced this on page 10, as well as captions of figure 4, and figure S3:

*“Since the strength of antibiotic is a parameter that exhibits significant variability (5-fold differences in decay rates; S2 Table), a stronger dose was simulated which demonstrated comparable results at both the 12 and 24 week mark for the placebo arm (S3 Fig).”*

It would be great to hear more discussion about mechanistically "why" the pre-treatment + probiotics work better than just probiotics. It's common practice to wipe out the microbiome first including the pathogen and then properly build it up. Authors start this paragraph "For probiotic regimens that include pre-treatment with antimicrobials like metronidazole of clindamycin, the impact of nAB on the probiotic strain becomes less important and the impact of endogenous Lactobacillus spp. is more prominent" but then this is actually not followed up properly. Antibiotics kill Lactobacillus spp. , give space to L. crispatus CTV-05 to do its job, and then Lactobacillus spp. grow back again? If so, does that mean we need to create a transient dysbiosis in the vaginal microbiome to allow L. crispatus CTV-05 dominance? What does this mean for colonization resistance against other infections in general?

The first line treatment for BV is metronidazole, which does not kill *Lactobacillus* spp. as these species are generally resistant to metronidazole. Thus, in this platform the simulated antibiotic does not deplete *Lactobacillus* spp., only nAB. In some scenarios, the probiotic strain (*L. crispatus CTV-05)* can stably integrate into the community and in other cases it can facilitate the growth of endogenous *Li* or oLB.

We demonstrate these results in Figure 5 where we assess which parameters were most associated with each possible response type according to treatment regimens.

In Fig. 5, where we compared the short-term (no ABX) with the Lactin-V regimen. Using the scores and loadings plot in the PLS-DA model, we identified that in the short-term regimen, _αnAB->P_ is the most important parameter contributing to the separation of nAB-dominant responses from other optimal responses. In contrast the Lactin-V regimen (MTZ+probiotic), the two most important parameters that distinguish treatment failure (primary nAB dominant responses across the virtual population) are α_P->Li_ and α_Li->P_.

We hypothesize that because nAB is selectively reduced, whichever endogenous *Lactobacillus* spp. that remains is going to be important for how the probiotic species impacts the community in the long-term. This result aligns with a report that endogenous *Lactobacillus* spp. can impact probiotic (*L. crispatus* CTV-05) efficacy [19]. In this virtual population platform, patient communities are more frequently mixed nAB with Li over nAB with oLB, which is why the interaction terms with Li exhibit higher magnitude loadings in the PLSDA.

To better follow up this we added this in the paragraph in Discussion on page 14:

*“For probiotic regimens that include pre-treatment with the first line BV antibiotic metronidazole, the impact of nAB on the probiotic strain becomes less important and the impact of endogenous Lactobacillus spp. is more prominent (α_Li→P_, α_oLB→P_). This result is because metronidazole only inhibits the nAB population, whereas Lactobacillus spp. are generally resistant [20]. Therefore pre-treatment with the antibiotic MTZ results in lower levels of nAB at the time of probiotic administration and a higher impact of endogenous Lactobacillus spp. that are present when probiotic is administered, which is more commonly Li in BV+ individuals and in this virtual patient population.”*

We removed clindamycin from the statement because clindamycin is not typically used to treat BV as long as individuals are responsive to MTZ. The reasoning is due to concerns that you noted in your response, where clindamycin can kill both nAB and *Lactobacillus* spp., resulting in a higher chance of opportunistic infections.

"This framework indicated for endogenous oLB to become dominant after treatment that probiotic strains must have a facilitative interaction with oLB (positive P->oLB), inhibitory interaction with Li (negative P->Li), and oLB must have a weak or inhibitory (negative oLB->P) interaction with the probiotic strain." -> "weak or inhibitory"? Is it weak "and" inhibitory? Also, I don't really understand how authors came to this conclusion quantitatively. Where in the results is this indicated? Say both P->oLB and oLB->P are negative, but P->oLB is smaller in magnitude. Wouldn't it still work? I am very sorry if I missed that plot, but couldn't see it.

We apologize for the confusion. This means that oLB must have a weak positive impact or a negative (inhibitory) impact on P. In other words, for endogenous oLB to dominate, α_oLB->P_ should not be a strong positive value. This is indicated by the results in Fig. S4C and S5C. We displayed the parameter distributions that correspond to each particular response type.

To clarify, we have rephrased it on page 14 as shown below:

*“This framework indicated for endogenous oLB to become dominant after treatment, probiotic strains must have a facilitative interaction with oLB (positive α_P→oLB_), inhibitory interaction with Li (negative α_P→Li_), and oLB must have a weak positive or inhibitory (negative α_oLB→P_) impact on the probiotic strain.”*

I think the end of the 4th paragraph of the discussion should be better organized in terms of ideas. Are you trying to say that you can have probiotics either directly inhibiting L. iners or indirectly inhibiting it by supporting its other endogenous competitors?

Thank you for pointing this out. We have now rephrased it to better justify how probiotics can either directly or indirectly inhibit L. iners. The revised paragraph is below:

*“A potential oLB-promoting probiotic strain could be one that selectively inhibits L. iners, such as the human intestinal Lactobacillus spp. strain (L. paragasseri K7) and a less potent vaginal strain (L. gasseri 105-1) [32]. Combinatorial therapy that selectively targets L. iners could potentially enhance a probiotic strain that may not inherently have selectivity against L. iners. Recent studies have suggested that targeting the cysteine dependence of L. iners could reduce competition between L. iners and oLB [31]. Overall, these findings further support the idea that increased characterization of the interactions between probiotic strains and endogenous Lactobacillus spp. could significantly improve probiotic design. Probiotic strategies can thus either directly inhibit L. iners or indirectly reduce its competitiveness through specific metabolic dependencies.”*

Methods
"For example, if a strain was 10% nAB-dominant, 20% Li-dominant, 60% oLB- dominant, and 10% P-dominant the strain would be classified as a oLB-promoting strain." -> It's not the strain that is dominant right, it's the distribution of results after 12mo (or whatever the time frame is) using this strain as the probiotics? This sentence should be more clear.

We have now tried to clarify this. We defined the response type for each of the 500 probiotic strains generated using Latin Hypercube Sampling (LHS) based on the results from testing each strain across 2000 virtual population. The response type was determined by the highest frequency observed. To classify the community state type (CST) for each of the 2000 samples, we used the VALENCIA method, a nearest centroid classifier commonly used clinically. For each of the 500 probiotic strains, the dominant response type was identified as the CST that occurred with the highest frequency across the population.

To make it clearer we rephrased it as below:

*“For example, by testing a probiotic strain on 2000 virtual samples, if 10% of patients become nAB-dominant, 20% Li-dominant, 60% oLB-dominant, and 10% P-dominant, the strain would be classified as a oLB-promoting strain.”*

Regarding the mathematical model: where are the death rates when antibiotics are applied? Do you reduce the growth rate?

When simulating the impact of antibiotics, we use an additional death term which is a first order term added to the differential equation for nAB: -kkill *nAB.

We have now made this more clear in the methods on page 16.

*“For simulations involving antibiotic treatment with metronidazole, the net growth rate was set to be negative (kkill) resulting in a first order decay rate based on values reported in Mayer et al. 2015 [68]. This parameter change was set to impact nAB group for the time period associated with duration of the antibiotic regimen.*

$$\frac{d[nAB]}{dt}= kkill\left[ nAB \right] + \alpha_{nAB\to nAB}\left[ nAB \right]\left[ nAB \right]+ \alpha_{Li\to nAB}\left[ nAB \right]\left[ Li \right]+ \alpha_{oLB\to nAB}\left[ nAB \right]\left[ oLB \right]+ \alpha_{P\to nAB}\left[ nAB \right]\left[ P \right]$$

“

"These seven states were related to clinical data using a nearest centroid classifier of the predicted relative abundances." What does this mean? Did you fit your data or determined the parameter distributions given these steady state values? If so, what is the fitting procedure? If not, does this mean that given the distributions you used, you observed that they are "related"? Or picked the related ones among your virtual population? Because later you say "The virtual patient population was selected to match the CST equilibrium behavior distribution pattern of the Human Microbiome Project Cohort (HMP) described in Lee et al. 2023." I am really confused about the methodology here.

The seven states refers to the seven equilibrium points of the system of ODEs which were classified into CSTs using a nearest centroid classifier.

We have consolidated the text on the virtual patient population generation so it is more clear. First, a very large virtual population (30,000 patients) is generated through latin hypercube sampling (LHS) which are then classified into different CST equilibrium behaviors through the nearest centroid classifier and assessment of multi-stability. Then from that large population, the 2000 virtual patients were selected to match the frequencies of each CST equilibrium behavior observed in the Human Microbiome Project (HMP) cohort [5]. For better understanding we rephrased and added more notes on page 16:

*“**To test the impact of a probiotic strain at the population level, a virtual patient population was generated using Latin Hypercube Sampling of physiologically defined parameter ranges as described in Lee et al. 2023(S1 Table). Briefly, a large collection of virtual patients was generated (30,000) and then resampled to match clinically observed CST equilibrium behavior distribution pattern of the Human Microbiome Project Cohort (HMP) described in Lee et al. 2023. The CST equilibrium behavior describes the stability in CST classification over time, where subjects that consistently exhibit the same CST are considered mono-stable (1SS) and those that switch between different CSTs are considered multi-stable (2SS). Model predictions were related to these CST equilibrium behaviors using a nearest centroid classifier of the predicted relative abundances, and whether the system exhibited mono or multiple equilibrium states referred to as CST equilibrium behavior. The centroids for the nearest centroid classifier were determined from VALENCIA as described in Lee et al. 2023 [1,2]. Probiotic strains were tested on the subset of 2,000 HMP cohort virtual patients that could obtain a nAB-dominant (BV positive) state at equilibrium which includes the 1SS nAB dominant (60%), 2SS nAB dominant / Li dominant (31%), and 2SS nAB dominant / Li dominant CST (9%) equilibrium behaviors. The HMP cohort had a similar frequency of self-identified White/Caucasian subjects relative to persons of color as the Lactin-V cohort (32% versus 35%, respectively) [41].”*

Supplementary

Figure S1 is a bit confusing, especially when the reader goes there right after the first paragraph of the results. This is in the supplementary anyway so you can afford to go a bit longer in the figure caption and make it easier to interpret.
In general, all figures in the main text and the supplementary should be standalone.

Thanks for the important point. We provided more information on the Figure S1 caption for easier interpretation:

*“In this framework, we tested various hypothetical probiotic strains, focusing on their interaction parameters with endogenous species. Additionally, we simulated our model using different probiotic and antibiotic regimens. Through these simulations, we identified the most important probiotic design criteria to address the issue of recurrent BV.”*

**References for the Reviewer Response:**

1.	France MT, Ma B, Gajer P, Brown S, Humphrys MS, Holm JB, et al. VALENCIA: a nearest centroid classification method for vaginal microbial communities based on composition. Microbiome. 2020;8: 166. doi:10.1186/s40168-020-00934-6

2.	Lee CY, Diegel J, France MT, Ravel J, Arnold KB. Evaluation of vaginal microbiome equilibrium states identifies microbial parameters linked to resilience after menses and antibiotic therapy. PLOS Computational Biology. 2023;19: e1011295. doi:10.1371/journal.pcbi.1011295

3.	Ravel J, Gajer P, Abdo Z, Schneider GM, Koenig SSK, McCulle SL, et al. Vaginal microbiome of reproductive-age women. PNAS. 2011;108: 4680–4687. doi:10.1073/pnas.1002611107

4.	Gajer P, Brotman RM, Bai G, Sakamoto J, Schütte UME, Zhong X, et al. Temporal Dynamics of the Human Vaginal Microbiota. Sci Transl Med. 2012;4: 132ra52. doi:10.1126/scitranslmed.3003605

5.	Ravel J, Brotman RM, Gajer P, Ma B, Nandy M, Fadrosh DW, et al. Daily temporal dynamics of vaginal microbiota before, during and after episodes of bacterial vaginosis. Microbiome. 2013;1: 1–6. doi:10.1186/2049-2618-1-29

6.	Witkin SS, Mendes-Soares H, Linhares IM, Jayaram A, Ledger WJ, Forney LJ. Influence of vaginal bacteria and D- and L-lactic acid isomers on vaginal extracellular matrix metalloproteinase inducer: implications for protection against upper genital tract infections. mBio. 2013;4: e00460-13. doi:10.1128/mBio.00460-13

7.	Amabebe E, Anumba DOC. Mechanistic Insights into Immune Suppression and Evasion in Bacterial Vaginosis. Curr Microbiol. 2022;79: 84. doi:10.1007/s00284-022-02771-2

8.	Edwards VL, Smith SB, McComb EJ, Tamarelle J, Ma B, Humphrys MS, et al. The Cervicovaginal Microbiota-Host Interaction Modulates Chlamydia trachomatis Infection. mBio. 2019;10. doi:10.1128/mBio.01548-19

9.	McKay MD, Beckman RJ, Conover WJ. A Comparison of Three Methods for Selecting Values of Input Variables in the Analysis of Output from a Computer Code. Technometrics. 1979;21: 239–245. doi:10.2307/1268522

10.	Cohen CR, Wierzbicki MR, French AL, Morris S, Newmann S, Reno H, et al. Randomized Trial of Lactin-V to Prevent Recurrence of Bacterial Vaginosis. New England Journal of Medicine. 2020;382: 1906–1915. doi:10.1056/NEJMoa1915254

11.	Joseph RJ, Ser H-L, Kuai Y-H, Tan LT-H, Arasoo VJT, Letchumanan V, et al. Finding a Balance in the Vaginal Microbiome: How Do We Treat and Prevent the Occurrence of Bacterial Vaginosis? Antibiotics (Basel). 2021;10: 719. doi:10.3390/antibiotics10060719

12.	Atassi F, Brassart D, Grob P, Graf F, Servin AL. Lactobacillus strains isolated from the vaginal microbiota of healthy women inhibit Prevotella bivia and Gardnerella vaginalis in coculture and cell culture. FEMS Immunol Med Microbiol. 2006;48: 424–432. doi:10.1111/j.1574-695X.2006.00162.x

13.	Borgogna J-LC, Shardell MD, Grace SG, Santori EK, Americus B, Li Z, et al. Biogenic Amines Increase the Odds of Bacterial Vaginosis and Affect the Growth and Lactic Acid Production by Vaginal Lactobacillus spp. Appl Environ Microbiol. 2021 [cited 10 Mar 2021]. doi:10.1128/AEM.03068-20

14.	Chetwin E, Manhanzva MT, Abrahams AG, Froissart R, Gamieldien H, Jaspan H, et al. Antimicrobial and inflammatory properties of South African clinical Lactobacillus isolates and vaginal probiotics. Scientific Reports. 2019;9: 1917. doi:10.1038/s41598-018-38253-4

15.	Stein RR, Bucci V, Toussaint NC, Buffie CG, Rätsch G, Pamer EG, et al. Ecological modeling from time-series inference: insight into dynamics and stability of intestinal microbiota. PLoS Comput Biol. 2013;9: e1003388. doi:10.1371/journal.pcbi.1003388

16.	Kraeutler MJ, Soltis AR, Saucerman JJ. Modeling cardiac β-adrenergic signaling with normalized-Hill differential equations: comparison with a biochemical model. BMC Syst Biol. 2010;4: 1–12. doi:10.1186/1752-0509-4-157

17.	Mayer BT, Srinivasan S, Fiedler TL, Marrazzo JM, Fredricks DN, Schiffer JT. Rapid and Profound Shifts in the Vaginal Microbiota Following Antibiotic Treatment for Bacterial Vaginosis. J Infect Dis. 2015;212: 793–802. doi:10.1093/infdis/jiv079

18.	Petrina MAB, Cosentino LA, Rabe LK, Hillier SL. Susceptibility of bacterial vaginosis (BV)-associated bacteria to secnidazole compared to metronidazole, tinidazole and clindamycin. Anaerobe. 2017;47: 115–119. doi:10.1016/j.anaerobe.2017.05.005

19.	Antonio MAD, Meyn LA, Murray PJ, Busse B, Hillier SL. Vaginal colonization by probiotic Lactobacillus crispatus CTV-05 is decreased by sexual activity and endogenous Lactobacilli. J Infect Dis. 2009;199: 1506–1513. doi:10.1086/598686

20.	Petrina MAB, Cosentino LA, Rabe LK, Hillier SL. Susceptibility of bacterial vaginosis (BV)-associated bacteria to secnidazole compared to metronidazole, tinidazole and clindamycin. Anaerobe. 2017;47: 115–119. doi:10.1016/j.anaerobe.2017.05.005

**Have the authors made all data and (if applicable) computational code underlying the findings in their manuscript fully available?**
The [PLOS Data policy](https://c05y1x9s.r.us-east-2.awstrack.me/L0/https:%2F%2Fjournals.plos.org%2Fploscompbiol%2Fs%2Fmaterials-and-software-sharing/1/010f019133add60d-5dc736b1-fdf4-4a00-bfce-1d27fd37e55b-000000/Tfmo3j_d66n_a7HLMPc8dchDQ-Q=171) requires authors to make all data and code underlying the findings described in their manuscript fully available without restriction, with rare exception (please refer to the Data Availability Statement in the manuscript PDF file). The data and code should be provided as part of the manuscript or its supporting information, or deposited to a public repository. For example, in addition to summary statistics, the data points behind means, medians and variance measures should be available. If there are restrictions on publicly sharing data or code —e.g. participant privacy or use of data from a third party—those must be specified.

Reviewer #1: Yes

Reviewer #2: Yes

Reviewer #3: Yes

PLOS authors have the option to publish the peer review history of their article ([what does this mean?](https://c05y1x9s.r.us-east-2.awstrack.me/L0/https:%2F%2Fjournals.plos.org%2Fploscompbiol%2Fs%2Feditorial-and-peer-review-process%23loc-peer-review-history/1/010f019133add60d-5dc736b1-fdf4-4a00-bfce-1d27fd37e55b-000000/_xPtilW0TyyucccWso8EuZkSBZc=171)). If published, this will include your full peer review and any attached files.


If you choose “no”, your identity will remain anonymous but your review may still be made public.


**Do you want your identity to be public for this peer review?** For information about this choice, including consent withdrawal, please see our [Privacy Policy](https://c05y1x9s.r.us-east-2.awstrack.me/L0/https:%2F%2Fwww.plos.org%2Fprivacy-policy/1/010f019133add60d-5dc736b1-fdf4-4a00-bfce-1d27fd37e55b-000000/yG5fMYRCYhGw6nCWuEdcVWtEaHk=171).

Reviewer #1: No

Reviewer #2: No

Reviewer #3: No

Figure Files:

While revising your submission, please upload your figure files to the Preflight Analysis and Conversion Engine (PACE) digital diagnostic tool, [https://pacev2.apexcovantage.com](https://c05y1x9s.r.us-east-2.awstrack.me/L0/https:%2F%2Fpacev2.apexcovantage.com%2F/1/010f019133add60d-5dc736b1-fdf4-4a00-bfce-1d27fd37e55b-000000/6La74KlZ56MZLfEsCaNAZ9dn67I=171). PACE helps ensure that figures meet PLOS requirements. To use PACE, you must first register as a user. Then, login and navigate to the UPLOAD tab, where you will find detailed instructions on how to use the tool. If you encounter any issues or have any questions when using PACE, please email us at [figures@plos.org](mailto:figures@plos.org).

Data Requirements:

Please note that, as a condition of publication, PLOS' data policy requires that you make available all data used to draw the conclusions outlined in your manuscript. Data must be deposited in an appropriate repository, included within the body of the manuscript, or uploaded as supporting information. This includes all numerical values that were used to generate graphs, histograms etc.. For an example in PLOS Biology see here: <http://www.plosbiology.org/article/info%3Adoi%2F10.1371%2Fjournal.pbio.1001908#s5>.

Reproducibility:

To enhance the reproducibility of your results, we recommend that you deposit your laboratory protocols in [protocols.io](http://protocols.io/), where a protocol can be assigned its own identifier (DOI) such that it can be cited independently in the future. Additionally, PLOS ONE offers an option to publish peer-reviewed clinical study protocols. Read more information on sharing protocols at [https://plos.org/protocols?utm_medium=editorial-email&utm_source=authorletters&utm_campaign=protocols](https://c05y1x9s.r.us-east-2.awstrack.me/L0/https:%2F%2Fplos.org%2Fprotocols%3Futm_medium=editorial-email%26utm_source=authorletters%26utm_campaign=protocols/1/010f019133add60d-5dc736b1-fdf4-4a00-bfce-1d27fd37e55b-000000/Biwzh8w5JLTwQvWH5sN6JHf9tzs=171)
